# Treatment and prognosis of spinal cord reperfusion injury after cervical spinal canal stenosis surgery: a case report

**DOI:** 10.3389/fsurg.2026.1675871

**Published:** 2026-02-06

**Authors:** Wenbo Diao, Jiankun Yang, Ya-nan Hu, Caili Lou

**Affiliations:** Department of Spinal Neurosurgery, Zhoukou Orthopedic Hospital, Zhoukou, Henan, China

**Keywords:** cervical spinal canal stenosis, corticosteroid pulse therapy, reperfusion injury, spinal cord edema, surgical treatment

## Abstract

**Background:**

Spinal cord reperfusion injury of the cervical spine is a rare but severe postoperative complication, typically occurring after the decompression of chronically compressed spinal cord tissue. The report aims to present a case of early postoperative spinal cord reperfusion injury that was successfully managed, and to discuss its underlying pathogenesis, treatment strategies, and clinical outcomes.

**Case report:**

A 63-year-old middle-aged male was admitted with a 3-month history of neck and shoulder pain accompanied by numbness and pain in both upper limbs. MRI and clinical evaluations revealed cervical spinal canal stenosis with radiculopathy. The patient underwent anterior cervical discectomy and fusion (ACDF), with no intraoperative complications noted. 2 h after the operation, when the patient regained consciousness, it was found that the muscle strength of both lower limbs was grade 1, that of both upper limbs was grade 2, and the skin sensation of the lower limbs was gradually fades. However, four hours after surgery, upon regaining consciousness, the patient developed complete quadriplegia and loss of skin sensation, with progressive worsening. An emergency MRI ruled out intracranial pathology but revealed spinal cord edema at the surgical site. Based on the clinical course and imaging findings, spinal cord reperfusion injury was suspected. The patient was immediately transferred through the emergency “green channel” for urgent posterior cervical laminoplasty to achieve expanded decompression, accompanied by intraoperative and postoperative high-dose corticosteroid therapy. One day after the second surgery, the patient's muscle strength improved to Grade 3. Following two months of postoperative treatment and rehabilitation, the patient made a full recovery and was discharged. Follow-up MRI demonstrated substantial resolution of spinal cord edema and restoration of spinal cord morphology. This case illustrates that early recognition of spinal cord reperfusion injury and timely, appropriate intervention can significantly improve neurological outcomes, providing valuable insight for the management of similar cases.

**Conclusion:**

Spinal cord reperfusion injury after cervical spine surgery is rare, but once it occurs, it requires a high level of clinical vigilance. Identifying the underlying cause, making a rapid diagnosis, and initiating timely surgical intervention combined with corticosteroid pulse therapy are essential to preventing irreversible neurological damage.

## Introduction

Cervical spinal canal stenosis (CSCS) refers to a condition in which the volume of the cervical spinal canal is reduced due to various factors, such as degenerative changes, intervertebral disc herniation, ossification of the posterior longitudinal ligament, hypertrophy of the ligamentum flavum, and osteophyte formation (1). This reduction leads to chronic spinal cord compression and results in neurological dysfunction. Clinically, CSCS is characterized by symptoms such as numbness and weakness in the limbs, gait instability, and impaired fine motor skills (2, 3). In severe cases, it can progress to spastic paralysis and dysfunction of bladder and bowel control. Surgical decompression remains an effective treatment for patients with moderate to severe cervical canal stenosis, as it can significantly relieve symptoms and slow disease progression. However, recent reports have noted that some patients experience early postoperative neurological deterioration, manifested by decreased muscle strength or even paralysis.

Despite the absence of conventional complications such as hematoma or mechanical compression on postoperative imaging, MRI often reveals intramedullary T2 hyperintensity, suggesting the presence of spinal cord reperfusion injury (SCRI). Preoperative neurological function assessment and imaging evaluation of cervical spinal cord injury play a crucial role in the formulation of surgical plans by surgeons, postoperative neurological recovery of patients, and the reduction of the occurrence of postoperative complications (4, 5). In addition, the stability of intraoperative blood pressure measurement can effectively protect the blood supply to the spinal cord, reduce intraoperative bleeding, and decrease the occurrence of complications.

Reperfusion injury refers to a pathological process in which previously ischemic tissues suffer secondary damage upon the restoration of blood flow, due to factors such as excessive production of reactive oxygen species, activation of inflammatory mediators, and disruption of the blood–spinal cord barrier (6, 7). While reperfusion injury has been extensively studied in organs such as the heart, kidneys, and brain, it remains relatively rare in the context of cervical spine surgery. If not promptly recognized and treated, cervical spinal cord reperfusion injury can lead to severe outcomes, including irreversible paralysis. This paper presents a case of SCRI following surgery for CSCS, and systematically discusses its diagnosis, management, and recovery process, aiming to provide a reference for the treatment of similar cases.

## Case presentation

A 63-year-old male patient was admitted with a 3-month history of neck and shoulder pain accompanied by numbness and pain in both upper limbs. Physical examination on admission revealed mild tenderness over the spinous processes of C3–C7 and positive tenderness in the bilateral scapular regions. Sensory examination showed significantly reduced pain and light touch sensation in both upper limbs, with numbness upon pinprick testing. Muscle strength in the right deltoid, biceps, and triceps was graded as 3+, and 4 in the corresponding muscles on the left. Bilateral hand grip strength was preserved. Brachial plexus stretch tests and foraminal compression tests were positive on both sides.

**Figure 1:**
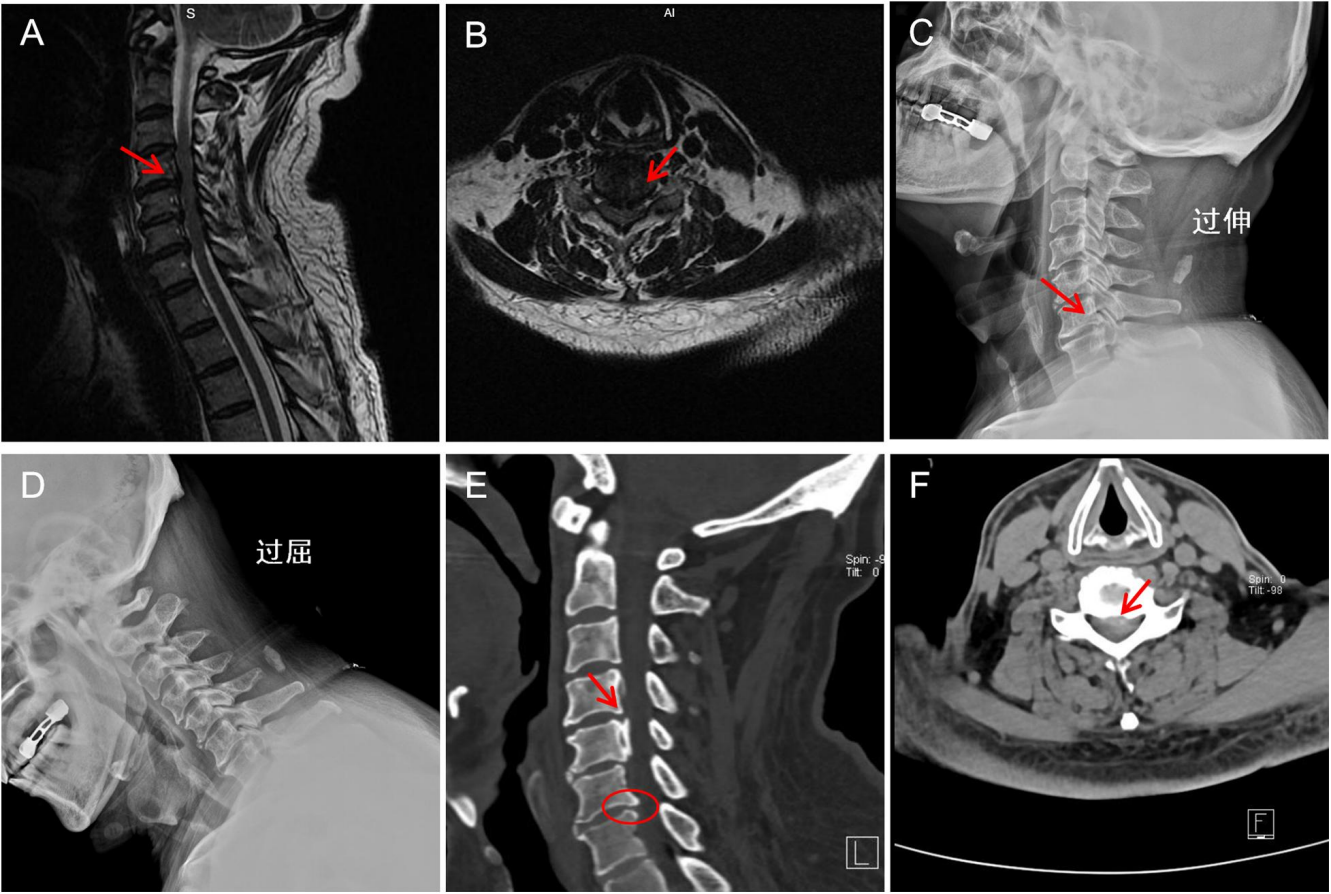
Preoperative imaging examinations of the patient. **(A)** Sagittal MRI showing cervical spinal canal stenosis with hypertrophy and proliferation of the posterior longitudinal ligament and ligamentum flavum. **(B)** Axial MRI image showing significant spinal cord compression. **(C)** Extension-position dynamic radiograph showing straightening of the normal cervical lordosis and osteophyte formation at the posterior margin of the C6/7 vertebral body. **(D)** Flexion-position dynamic radiograph revealing cervical instability. **(E)** CT image showing ossification of the posterior longitudinal ligament at the C4 and C5 vertebral levels. **(F)** CT image showing disc herniation at C4–C6 with spinal cord compression.

Magnetic resonance imaging (MRI) demonstrated cervical spinal canal stenosis at the C3–C6 levels, with hypertrophy and proliferation of the ligamentum flavum, as well as intervertebral disc herniation from C3 to C7 (Figures 1A,B). Further evaluation with dynamic radiographs (DR) and computed tomography (CT) was performed. DR revealed a straightened cervical physiological curvature and osteophyte formation at the C6/7 level (Figures 1C,D). CT imaging showed ossification of the posterior longitudinal ligament (OPLL) at C4 and C5, with disc herniation at C4/5 and C5/6 compressing the spinal cord (Figures 1E,F).

**Figure 2:**
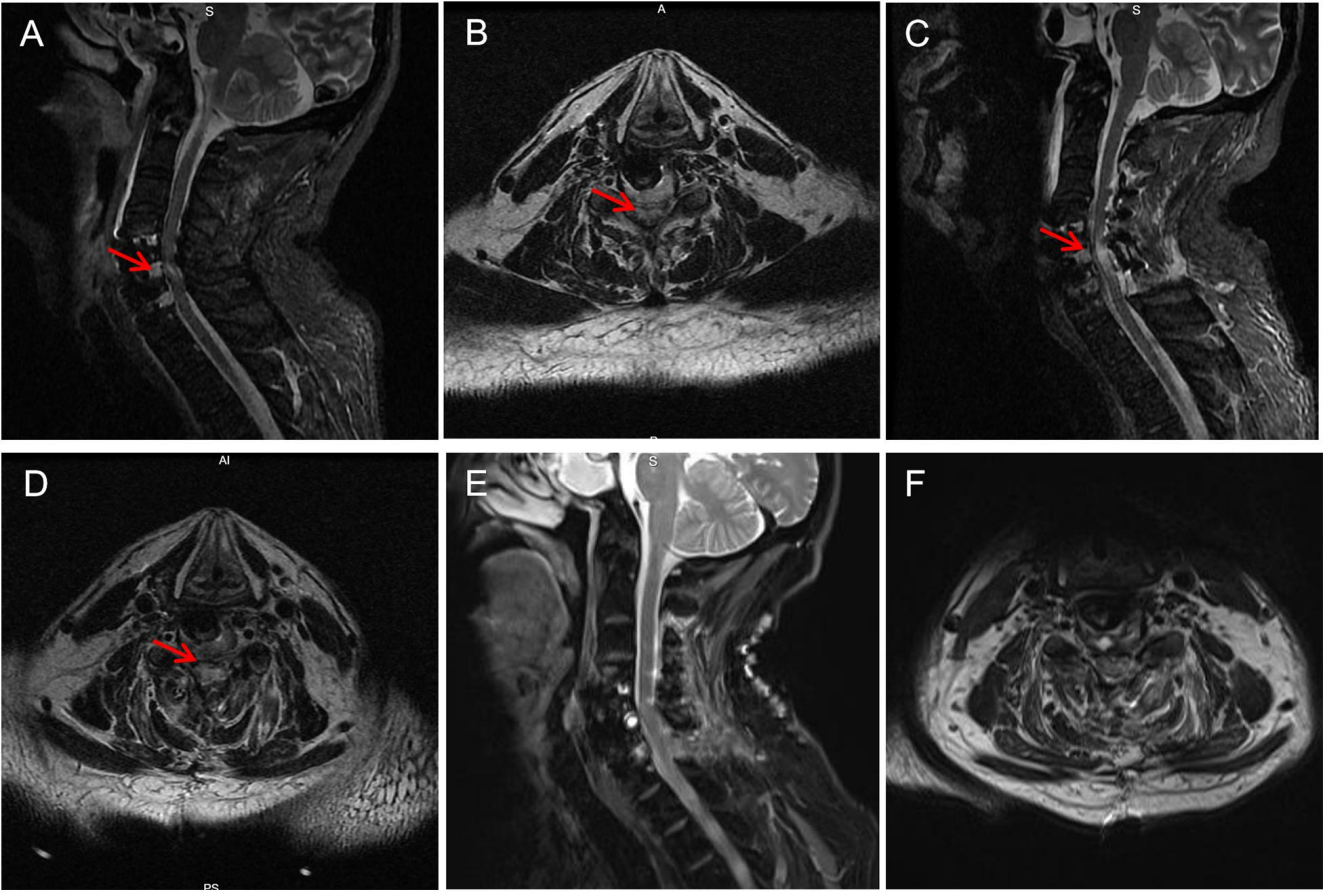
The patient underwent follow-up imaging examination after cervical spine surgery. **(A)** The sagittal plane of MRI shows high signal in the spinal cord part of C5-6,The physiological curvature of the cervical vertebrae is overextended; **(B)** the aqueous plane of MRI shows high signal in the spinal cord part of C5-6; **(C)** the sagittal plane of MRI shows a large amount of high signal in the C5-6 spinal cord, and the physiological curvature of the cervical vertebrae has partially recovered; **(D)** the horizontal MRI plane shows a large area of high signal in the C5-6 spinal cord; **(E)** the sagittal plane of MRI shows that the high signal of C5-6 spinal cord disappears; **(F)** the abnormal signal of the horizontal spinal cord in the C5surgical segment disappeared.

These findings confirmed the diagnosis of cervical spinal canal stenosis.ased on the preoperative imaging findings, the patient underwent an anterior cervical decompression and discectomy. The surgery proceeded smoothly. However, two hours postoperatively, after regaining consciousness, the patient was found to have decreased muscle strength in all four limbs: muscle strength in both lower limbs was grade 1, and in both upper limbs was grade 2, and the skin sensation of the lower limbs was gradually fades. Emergency cranial and cervical MRI scans were performed to exclude intracranial pathology. Through MRI imaging examinations of the head and neck, we ruled out the occurrence of cranial hemorrhage and cerebral infarction, epidural hematoma of the cervical spinal cord, residual compression, dislocation of grafts or implants, intraoperative or postoperative hypotension/hypoxia, or position-related cervical hyperextension injury. However, based on the T2-weighted MRI images of the cervical spinal cord showing significant edema in the C5-6 segment, accompanied by cervical hyperextension deformity (Figures 2A,B), it is suggested that it might be SCRI. We consider that the patient's cervical spinal cord edema is related to cervical hyperextension caused by the patient's body position. Cervical spinal cord MRI T2-weighted images revealed significant edema at the C5-6 segment, accompanied by cervical hyperextension deformity (Figures 2A,B), raising suspicion of SCRI. Four hours after surgery, upon regaining consciousness, the patient developed complete quadriplegia (muscle strength Grade 0) and loss of skin sensation, with progressive worsening.

The patient was immediately treated with high-dose methylprednisolone succinate pulse therapy (The preoperative dosage is 20 mg/kg, completed within 30 min, with a total dose of 1,500 mg. The intraoperative dose was 5 mg/kg, and the total dose was 800 mg. After the operation, the patient was continued to be treated with 200 mg of methylprednisolone sodium succinate once a day for three days). Simultaneously, an emergent posterior cervical unilateral “open-door” decompression was performed via a green channel. Two hours after the second posterior cervical spine surgery, upon awakening, the patient's lower limb muscle strength improved to grade 3−, and skin sensation partially recovered. The patient continued receiving high-dose methylprednisolone pulse therapy and, once stabilized, was transferred to rehabilitation for adjunctive therapies including acupuncture, neuromuscular electrical stimulation, and therapeutic massage. A follow-up MRI three days postoperatively showed marked spinal cord edema at the C5–C6 segments (Figures 2C,D).

At the two-month follow-up, MRI demonstrated complete resolution of the cervical spinal cord edema at the C5–C6 segments (Figures 2E,F). The patient's muscle strength and skin sensation had fully recovered, achieving a good functional outcome and was discharged with satisfactory rehabilitation progress.

## Discussion

SCRI after cervical spine surgery is a rare but serious complication, primarily occurring when a chronically compressed spinal cord is suddenly decompressed, leading to secondary neurological deterioration related to the restoration of blood flow. Clinically, SCRI manifests as a significant decline in motor and sensory functions within a short time after surgery, sometimes progressing to complete paralysis.

In this case, the patient had preoperative spinal cord compression and developed decreased muscle strength in all four limbs postoperatively. MRI revealed diffuse spinal cord edema, leading to a diagnosis of reperfusion injury. Following emergency surgery and comprehensive treatment, the patient showed significant improvement, suggesting that this condition may be potentially reversible. This case holds important clinical value, providing new insights into spinal cord reperfusion injury following cervical spinal canal stenosis surgery, and helps clinicians deepen their understanding of this complication. Given the scarcity of related case reports, this case warrants heightened attention from clinicians.

The research indicates that the pathogenesis of spinal cord reperfusion injury after cervical spinal canal decompression is mainly related to oxidative stress, inflammatory mediator release, and apoptosis occurring during the ischemia-reperfusion process (8). Studies have proposed that the sudden restoration of blood flow to a chronically compressed spinal cord leads to massive reactive oxygen species generation, triggering lipid peroxidation reactions that damage cell membrane structure and function, thereby causing spinal cord edema and neurological injury. Additionally, the release of inflammatory mediators further exacerbates spinal cord damage (9). The use of glucocorticoid spinal cord in cervical spinal cord injury is mainly based on its anti-inflammatory, immunosuppressive, antioxidant and cell-protective effects. By reducing the initial inflammatory response and cellular damage, the glucocorticoids help optimize the environment during the nerve repair process. However, long-term and high-dose use of glucocorticoids cannot effectively improve the neurological function of patients with spinal cord injury; instead, it increases the occurrence of adverse events such as gastrointestinal bleeding or severe infections. Fortunately, no related complications occurred in our report.

This case report conveys several important messages. First, preoperative positioning and endotracheal intubation under general anesthesia must fully consider their impact on the spinal cord to avoid reperfusion injury caused by improper handling. Clinicians should pay close attention to these details and implement appropriate measures, such as maintaining the physiological cervical curvature during positioning and avoiding excessive head extension during intubation, to reduce the risk of complications. Second, once spinal cord edema secondary to reperfusion injury is identified, effective and timely intervention is essential. Emergency surgery to relieve spinal cord edema can rapidly reduce spinal cord compression and create favorable conditions for neurological recovery. High-dose corticosteroid pulse therapy administered intraoperatively and postoperatively can effectively eliminate or alleviate spinal cord edema by suppressing inflammation and reducing oxidative stress damage.

Posterior cervical decompression surgery has the following advantages in treating cervical spinal cord injury (10–12): (1) it can directly relieve compression from the posterior side of the spinal cord; (2) after decompression, the spinal cord floats posteriorly, indirectly reducing the compression at the anterior side of the spinal canal; (3) by removing the lamina, the spinal cord can be moved backward to relieve anterior compression, reduce the arterial and venous tension on the spinal cord surface, and promote blood supply recovery; (4) it offers more complete decompression of the spinal canal. Therefore, when cervical spinal cord edema occurs due to anterior cervical injury, selecting posterior cervical decompression can effectively alleviate further worsening of spinal cord edema by expanding the spinal canal volume.

Finally, the good prognosis observed in this patient—muscle strength recovery and resolution of spinal cord edema—provides a successful treatment experience for similar cases. In future clinical practice, physicians should enhance their awareness of spinal cord reperfusion injury after cervical spinal canal stenosis surgery, strengthen preoperative assessment and intraoperative monitoring, and promptly detect and manage complications to improve patient outcomes.

## Conclusion

Although SCRI after cervical decompression surgery is rare, it requires heightened vigilance once it occurs. Identifying the underlying cause, making a rapid diagnosis, and initiating timely surgical intervention combined with corticosteroid pulse therapy are key to preventing irreversible neurological damage. Proper patient positioning before surgery and careful intraoperative management help reduce the risk of this complication.

## Data Availability

The original contributions presented in the study are included in the article/Supplementary Material, further inquiries can be directed to the corresponding author.
