# Supplementary material for: Treatment and prognosis of spinal cord reperfusion injury after cervical spinal canal stenosis surgery: a case report
Source: Front Surg. 2026 Feb 6;13:1675871. doi: 10.3389/fsurg.2026.1675871 (PMC12920454; doi:10.3389/fsurg.2026.1675871)
Supplement: Supplementary file 1 [file Datasheet1.pdf]

Supplementary attachment

Schedule of neurological function changes before and after surgery

Time point	Clinical feature	Treatment
Pre-operation	The muscle strength score of the patient's right deltoid, biceps and triceps is 3+, and the muscle strength score of the corresponding left muscles is 4. The muscle strength scores of the right deltoid, biceps and triceps are 3+, while those of the corresponding muscles on the left are 4.	Preparing an operation
During operation	The patient is under anesthesia and the blood pressure measurement is within the normal range. Ventilator-assisted breathing, stable condition; The blood oxygen test is normal.	Surgical treatment
0 hours (immediate post-op)	The patient is in a coma and is assisted with breathing by a ventilator. Lack of autonomous consciousness.	Observe the patient's state of consciousness
2 hours (post-operation)	The patient's self-awareness has been restored, and has resumed spontaneous breathing. The skin sensation in both lower limbs has decreased, and the muscle strength of all limbs has declined. The muscle strength of both upper limbs is grade 2, and that of both lower limbs is grade 1.	Magnetic resonance imaging (MRI) was conducted in the surgical area of the head and neck to assess any abnormalities in the surgical area and to evaluate abnormal results such as cranial hemorrhage and infarction.

4 hours<br>(post-operation)	The patient is conscious, but has lost sensation in both lower limbs and has zero muscle strength in the limbs.	The patient was prepared for the second operation, and at the same time, high-dose methylprednisolone hormone shock therapy was given to the patient before the operation.
Two hours after the second posterior cervical spine surgery	The patient is conscious, the skin sensation of both lower limbs has recovered, and the muscle strength of both upper limbs is grade 3. The muscle strength of both lower limbs is grade 3.	Observe the patient's state of consciousness.
2months<br>(post-operation)	The patient's limb muscle strength has returned to normal, and the skin sensation has also。returned to normal.	During the patient's hospitalization, traditional Chinese medical treatments such as acupuncture, neuromuscular electrical stimulation and therapeutic massage were provided to the patient。
